# PSORIASIS AND CARDIOMYOPATHY: AN INTRIGUING ASSOCIATION

**DOI:** 10.4103/0019-5154.70689

**Published:** 2010

**Authors:** Anupam Prakash, Deepshikha

**Affiliations:** *From the Department of Medicine, Era’s Lucknow Medical College & Hospital, Lucknow. U. P. India*

**Keywords:** *Psoriasis*, *dilated cardiomyopathy*, *heart failure*, *cardiac disorder*

## Abstract

A 25-year-old male symptomatic of heart disease for four months presented with biventricular failure. Echocardiography revealed dilated cardiomyopathy. He had skin lesions for 10 years which were clinically and histopathologically identified as psoriasis. Association of cardiomyopathy with psoriasis is uncommon and intriguing. The link between dilated cardiomyopathy and psoriasis on a common inflammatory background is discussed.

## Case Report

A 25-year-old male presented with progressive breathlessness, cough, pain of right upper abdomen and anorexia of four months duration. There was no history of fever, expectoration, chest pain, palpitations, syncope, joint pains, joint swelling, photosensitivity and no other complaints attributable to the nervous or abdominal system. Remarkably, he had a rash over the trunk and limbs for four months as well. He reported similar skin lesions over the last 10 years, occurring over time and responding transiently to local therapy, details of the therapy were not known to the patient. He was a smoker for last two years, smoking on an average 10 bidis a day. There was no other addiction. There was no family history of heart disease or sudden death or psoriasis.

**Figure 1:**
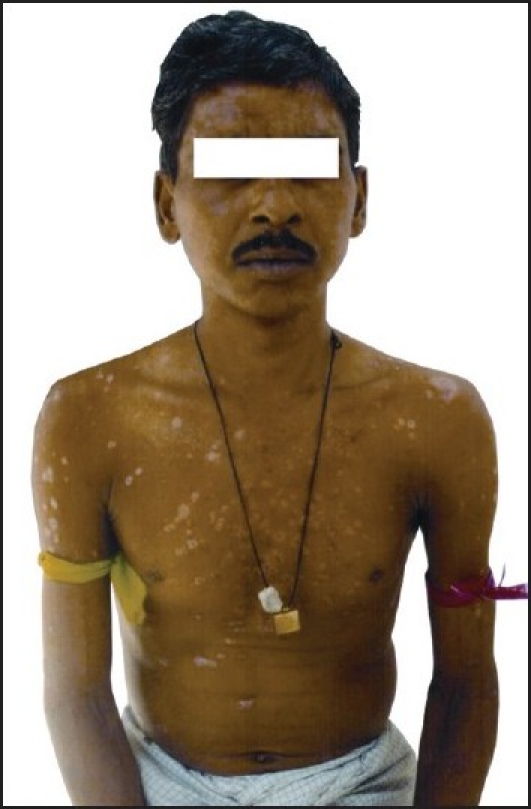
Typical skin lesions of psoriasis and postinflammatory hypopigmented skin lesions

Physical examination revealed conscious, oriented but uncomfortable male who was tachypnoeic, dyspnoeic, orthopnoeic; had a temperature of 98.4°F, pulse- 102/min regular, all peripheral pulses were palpable, BP-110/80 mm Hg, JVP pressure = 13 cm water. He had multiple plaques over the whole body including the scalp, face, trunk, upper and lower limbs, which were covered with silvery scales on an erythematous base, sharply demarcated from the adjoining skin; along with postinflammatory hypopigmented skin lesions [Figure 1]. Auspitz’s sign was positive. There was no evidence of arthritis or nail/mucosal involvement.

Cardiovascular examination revealed soft first and second heart sounds, but there was no third or fourth heart sound and no rub/click/murmur. Respiratory examination was notable for bilateral fine crepts in lower half of chest and bilateral rhonchi were also heard. Tender hepatomegaly and bilateral pitting pedal oedema were present.

A clinical diagnosis of psoriasis with biventricular failure was entertained.

Patient was propped up, and put on oxygen inhalation, parenteral diuretics (furosemide 40 mg i.v. bid), oral enalapril 2.5 mg OD, aspirin 150 mg OD, clopidogrel 75 mg OD and oral omeprazole 20 mg OD.

Investigations revealed Hb of 12.4 g/dl, leucocyte count of 14.5 × 10^3^cu. mm; P83, L11, E4, M2. ESR was 50 mm at the end of first hour (Westergren), Peripheral smear showed normocytic normochromic erythrocytes with polymorphonuclear leucocytosis, platelets were adequate. Blood urea was 38 mg/dl, serum creatinine 0.74 mg/dl, serum bilirubin 1.01 mg/dl, SGPT 74 U/L. Serum total cholesterol was 113 mg/dl, HDL cholesterol 25 mg/dl, LDL cholesterol 74.6 mg/dl, triglycerides were 67 mg/dl. Serum electrolytes and TSH were normal, VDRL, ANA and Rheumatoid factor were negative, Antistreptolysin O antibody titre was < 200 U/L, C-reactive protein was positive (by latex agglutination), ELISA for HIV 1 and 2 and antiHCV antibodies was negative. Urine- routine and microscopic examination was unremarkable. Chest X-ray revealed gross cardiomegaly and ECG findings were suggestive of left axis deviation, biatrial enlargement, ST-T changes in all leads, normal rhythm and no pathological q waves. Mild hepatomegaly and minimal ascites were evident on abdominal sonogaphy. Skin biopsy showed the characteristic picture of psoriasis.

Echocardiography findings showed a dilated left ventricle and global hypokinesia, mild mitral regurgitation and left ventricular ejection fraction of 32%. No left ventricular clot was detected, and rest of the parameters were normal. An impression of dilated cardiomyopathy was made. Spirometry was normal.

Patient improved gradually and dose of enalapril was increased to 5 mg OD. For psoriasis, patient was started on topical steroids and coal tar preparations. Patient was eventually discharged after a fortnight on a reduced dose of furosemide 40 mg P.O. OD and enalapril 5 mg OD, aspirin and salt restriction. The diagnosis at discharge was psoriasis, dilated cardiomyopathy with anasarca.

## Discussion

Psoriasis is a common chronic inflammatory dermatologic disorder, but its aetiology is poorly understood. There is a genetic component present, 50% have positive family history and linkage to HLA Cw6, HLA DR7 is known. The skin lesions are characterised by infiltration with activated T cells. Cytokines from activated T cells elaborate growth factors that stimulate keratinocyte hyperproliferation. As it is a T cell mediated disorder, clinical trials are underway with agents attempting to suppress the “inflammation characteristic” of psoriasis targetting TNF-alpha and other proinflammatory cytokines, T cell activation and lymphocyte trafficking.

Recently, “inflammation characteristic” in cardiomyopathies has been unravelled and a distinct entity has been described: “Inflammatory cardiomyopathy” defined as myocarditis in association with cardiac dysfunction. This is in addition to the five major forms of cardiomyopathies: dilated, restrictive, hypertrophic, right ventricular and nonclassifiable cardiomyopathies.[1] A pivotal role for autoimmunity in dilated cardiomyopathy is supported by the presence of organ-specific autoantibodies, inflammatory infiltrates and proinflammatory cytokines. Autoantibodies and abnormal cytokine profiles have been witnessed in first degree relatives with asymptomatic left ventricular enlargement and the fact that familial occurrence of “dilated cardiomyopathy” is observed in 20-30% cases suggests involvement of disrupted humoral and cellular immunity early in the development of disease. Similar humoral and cellular immune dysregulation are seen in autoimmune diseases and the frequency of autoimmune disorders *viz*. juvenile diabetes mellitus, rheumatoid arthritis, thyroiditis, psoriasis and asthma is also high in first-degree relatives of subjects with dilated cardiomyopathy. Therefore, “dilated cardiomyopathy” shares genetic risk factors with other diseases of presumed autoimmune aetiology. It is hypothesised that the same multiple genes in combination with environmental factors lead to numerous different autoimmune diseases including dilated cardiomyopathy.[1]

It will be pertinent to mention that in a University dermatology practice in USA[2] 73% of 753 cases of psoriasis had co-morbid diagnosis, the most common of which were hypertension, dyslipidemia, diabetes mellitus and heart disease, although renal failure and hepatitis were reported to be least likely. However, an interesting article raised the possibility of an entity called “psoriatic nephropathy”.[3] Moreover, microalbuminuria has been reported to be the only welldocumented abnormality in renal tests in psoriasis vulgaris. It is also an accepted fact that microalbuminuria is an independent risk factor for cardiovascular morbidity and mortality. High plasma endothelin-1 and elevated plasma rennin activity are believed to contribute to high prevalence of hypertension and cardiovascular disease in psoriasis. Angiotensin II may also play a contributory role. It is a product of angiotensin converting enzyme (ACE) action and is believed to be an endogenous proinflammatory molecule. ACE is present in uterus, placenta, vascular tissue, heart, brain, adrenal cortex and kidney, leukocytes, alveolar macrophages, peripheral monocytes, neuronal cells, epididymal cells; and may thus have a role in atherosclerosis, congestive heart failure, cerebrovascular accidents, bipolar disorder, schizophrenia, dementia, Alzheimer’s disease, psoriasis, atopic and non-atopic dermatitis, eczema, several acute and chronic inflammatory diseases and cancer.[4] Psoriasis is a systemic inflammatory state that confers increased cardiovascular risk in addition to the traditional risk factors, besides elevated cardiovascular disease risk factors and various co-morbidities viz. heart disease, hypertension, diabetes, dyslipidemia and microalbuminuria have been consistently observed in psoriasis patients.[5] Psoriasis has also been linked to an increased prevalence of metabolic syndrome.[6] In fact, the role of inflammation and higher prevalence of subclinical atherosclerosis and endothelial dysfunction have been demonstrated in psoriatic arthritis patients.[7] Moreover, the recent description of inflammatory cardiomyopathies lends credence to the common underlying pathogenetic association of cardiomyopathy and psoriasis occurring in the same subject. A literature search highlights that the association of dilated cardiomyopathy and psoriasis in the same individual is not only intriguing but also rare. One case of psoriatic arthritis associated with dilated cardiomyopathy has been reported from Japan, but the cardiomyopathy was associated with Takayasu’s arteritis.[8] Another similar case of psoriasis with dilated cardiomyopathy has been reported and the review commented whether the link was more than mere coincidence.[9] This case was 48-year-old and developed cardiomyopathy 15 years after development of psoriasis and had psoriatic arthritis for 10 years. This review did report nine other cases of psoriasis with dilated cardiomyopathy on a retrospective analysis in their institute.[9] The present case is different in that he is much younger in age (25 years at age of development of cardiomyopathy), though he had psoriasis for 10 years but characteristically did not have any evidence of psoriatic arthritis. In fact, the development of dyspnoea was associated with a flare-up of his skin lesions. The possibility of a viral myocarditis with dilated cardiomyopathy as sequelae can not be entirely ruled out, though it is unlikely as there was no acute episode at onset of symptoms and the symptoms were progressive and developed insidiously. Moreover, the fulminant course demonstrable in viral cardiomyopathies (arrhythmias and conduction blocks) was lacking in this case. An endomyocardial biopsy can demonstrate viral persistence or evidence of myocardial inflammation and can be helpful in arriving at a final diagnosis. However, this case did not undergo an endomyocardial biopsy because of financial constraints and lack of such a facility at our centre.

Search for previous cases of “dyspnoea in psoriasis” published in literature brought to light four cases of ARDS in pustular and erythrodermic psoriasis wherein also proinflammatory cytokines were believed to be involved.[10] The common link between psoriasis and cardiomyopathy is also supported by the fact that interferon therapy is known to induce acute or chronic cardiovascular toxicity and also exacerbate psoriasis. Similarly, TNF-alpha is a key mediator in psoriasis and interestingly TNF-alpha levels are elevated in “congestive heart failure” as well.

Dilated cardiomyopathy in association with psoriasis is an interesting association and the link to psoriasis could prompt the diagnosis of an “inflammatory dilated cardiomyopathy” or a “psoriatic cardiomyopathy”. It will be overzealous to label this case as “psoriatic cardiomyopathy” however, it is necessary to highlight its possibility, and further research on inflammatory cardiomyopathies may justify the existence of this entity namely “psoriatic cardiomyopathy”. It is pertinent for each general practitioner, physician and dermatologist to be aware of this association.
